# Early experiences matter: a review of the effects of prenatal environment on offspring characteristics in poultry

**DOI:** 10.3382/ps/pev343

**Published:** 2015-11-27

**Authors:** L. M. Dixon, N. H. C. Sparks, K. M. D. Rutherford

**Affiliations:** * Animal Behavior and Welfare, Animal and Veterinary Science Research Group, SRUC, West Mains Rd, Edinburgh EH9 3JG, UK; † Avian Science Research Center, Animal and Veterinary Science, Research Group, SRUC, West Mains Rd, Edinburgh EH9 3JG, UK

**Keywords:** early experiences, prenatal environment, post-hatch performance, welfare

## Abstract

Early life experiences can be important in determining offspring phenotypes and may influence interaction with the environment and hence health, welfare, and productivity. The prenatal environment of poultry can be divided into the pre-lay environment and the egg storage/incubation environment, both of which can affect offspring outcomes. The ability to separate maternal and egg/incubation effects makes birds well suited to this type of research. There are many factors, including feeding and nutrition, environmental conditions, husbandry practices, housing system, social environment, infectious environment, and maternal health status, that can influence both the health and performance and behavior and cognition of the offspring. There are some aspects of the environments that can be changed to produce beneficial effects in the offspring, like addition of certain additives to feed or short changes in incubation temperatures, while other aspects should be avoided to reduce negative effects, such as unpredictable feeding and lighting regimens. Measures of offspring characteristics may prove to be a useful method of assessing parent stock welfare if known stressors result in predictable offspring outcomes. This has the advantage of assessing the parent environment without interfering with the animals and possibly affecting their responses and could lead to improved welfare for the animals.

## INTRODUCTION

The early life (prenatal and neonatal) period has been found to be extremely important in shaping animal phenotypes throughout life. Examples of how variation in experience at the very start of life can impact upon lifetime biology have been seen across a wide range of different animal groups, including mammals, fish, and birds (reviewed by Rutherford et al., 2012). Similarly the human fetal environment has been shown to have lasting effects on offspring phenotype with correlations being found between low birth weight and later ill health (Barker, 2004). For farmed species it is becoming increasingly apparent that the early life environment may be critical in determining how well individual animals cope with their postnatal environment and has consequential effects on farm performance and individual animal health and welfare. In this paper, we will use the definition of welfare that refers to an animal's state in relation to its environment and its ability to cope in that environment. Animals that fail to cope or have difficulty coping in an environment are said to have poor welfare (Broom, 1991). As prenatal experiences determine how well an animal fits with its postnatal environment, prenatal experiences can affect welfare.

In the case of avian species, both pre-lay and egg storage/incubation (referred to subsequently as “incubation”) periods can influence the post-hatch development and responses of individual birds. Factors in the prenatal environment have been shown to have lasting effects on how the offspring respond to their environment (reviewed by Lickliter, 2005; Henriksen et al., 2011). It has been demonstrated that in addition to genetic factors a range of maternal and early life environmental factors can influence development, physiology, and behavior of the progeny (e.g. Lickliter, 2005). For example, prenatal exposure to androgens can influence growth and activity levels in the chicks (e.g. Eisling et al., 2006) and incubator-hatched quail chicks respond selectively to the maternal calls of their own species and show attention biases that help lead them to the proximity of their mother (e.g. Heaton et al., 1978). This phenomenon has begun to receive more attention in the past few decades and has led to a variety of studies of the effects of prenatal environment on poultry.

Avian species’ prenatal environments differ from that of mammals and can be divided into two phases: pre-lay and incubation. Consequently the maternal environment and the incubation/egg environment can have independent and lasting effects on the offspring (Mousseau and Fox, 1998; Lay and Wilson, 2002; Henriksen et al., 2011). This characteristic makes birds useful in the study of early life environment as influences directly from the mother (e.g. nutrition levels or antibody deposition) and the environment (e.g. temperature, day length) can be separated (Lickliter, 2005). The short generation intervals of some avian species such as chickens and quail also make them ideally suited for research. As a consequence, there are a number of studies designed to manipulate the pre-lay or incubating environment to assess subsequent effects on progeny. Thus the purpose of this paper is to review how stressors applied to the maternal parent and embryo affect the health and welfare of the offspring. There have been studies comparing the genetic contributions of the paternal parent on, e.g. offspring BW (Dottavio et al., 2005) or immunity (Li et al., 2008), but the effects of stressors applied to the sire on offspring outcomes have received little attention and will not be covered here. Due to the disparate nature of literature relating to this topic, and to ensure a transparent and repeatable selection process, a systematic review (**SR**) approach was adopted (Arnott et al., 2012).

SR uses scientific review methods to minimize systematic and random errors (Cook et al., 1997) that may occur in a more traditional narrative review where the author may have pre-conceived opinions and biases regarding a topic (Sargeant et al., 2006). Specific search strategies and inclusion criteria are used in SR, eliminating selection bias and subjecting all studies to critical appraisal. Generally, SR uses a quantitative analysis approach, such as meta-analysis. However, as with the Arnott et al. (2012) review, this was considered unsuitable due to the broad nature of the review topic, and therefore a systematic, transparent search strategy that could be repeated by others and a protocol to identify suitable studies to include were developed. Once these studies were identified, a narrative approach was used due to the broad review questions that examined many outcomes and a relatively small number of appropriate studies to examine the results and formulate reasonable conclusions.

## MATERIALS AND METHODS

### Searches

The online database “ISI Web of Knowledge,” which searches simultaneously ISI Web of Science (1970 to present), MEDLINE (1950 to present), and BIOSIS Previews (1969 to present), was used to examine the literature. The search terms were combinations of words relating to prenatal and incubation stress. The final search string is given below (asterisks indicate where the electronic database looked for words beginning with stated letters; for example, chicken* was used to find references relating to the words “chicken” and “chickens”):

(prenatal or maternal or perinatal or gestation*) and (stress or program* or nutrition* or effect*) and (poultry or chicken* or chick* or hen or bird* or avian)

Additionally, detailed searches of several relevant journals were conducted (e.g. *Applied Animal Behavior Science, Poultry Science, Animal Welfare, British Poultry Science*), and any articles deemed relevant but missed in the database search were included. This was all conducted as per the methods described in Arnott et al. (2012).

### Search Results

The initial search was conducted in Web of Knowledge on 07 Dec 2009, with updates added after a repeated search using similar terms on 09 July 2011 and 16 Oct 2013. After removal of duplicates, the search yielded 3,165 references in both English and non-English languages. The references and any available abstracts were imported into the Reference Manager (Thomson Reuters, New York City, NY) bibliographic database for manipulation and were then classified as outlined below.

### Classification of Results

A general overview of the result classifications will be presented here as the procedures and categories have been used in another publication (please refer to Arnott et al. (2012) for a detailed explanation).

Initially the references were screened for relevance and those that were clearly irrelevant or were review articles were removed (2,830 irrelevant papers were removed, leaving 335). The remaining papers were examined in more detail and classified according to whether they contained maternal outcomes only (i.e. with no progeny measures) or whether they investigated aspects of maternal nutrition or applied some form of stressor to the hen prior to ovulation or the egg during incubation and then investigated offspring outcomes. Stressor here refers to any form of non-nutritional challenge with impacts on hen biology.

As an aim of this review was to identify pre-hatch hazards that affect offspring health and welfare, studies were classified according to the early life treatment applied. Early life hazards were classified under the following headings: Feeding Method and Nutrition, Environmental Parameters, Artificial Parameters, Husbandry Parameters, Housing System, Social Environment, Infectious Environment, and Maternal Health Status.

### Quality Assessment

A quality assessment of the studies was made as recommended by Sargeant et al., (2006), using the following protocol, which was largely adopted from the REFLECT statement (O'Connor et al., 2010). Any studies not meeting these standards were discarded (for full details see Arnott et al., 2012). The criteria used included:

1. Randomization: individuals randomly allocated to treatment groups
2. Treatment intervention: experimental treatment intervention clearly described
3. Control: use of a suitable control group
4. Sample size: use of a sample size of five experimental units or more (as recommended by Festings and Altmann, 2002)
5. Statistical methods: use of appropriate and clear statistical methods
6. Avoidance of data repetition: multiple publications reporting on same data
7. Exclusion of conference abstracts/proceedings

After quality assessment, one reference was identified as a conference proceeding and 5 references failed to pass sample size, statistical methods, and control criteria and were removed. In total, 108 articles proceeded to the final data extraction process of the SR.

## RESULTS

### Feeding Method and Nutrition

The nutritional status of the mother and of the egg during incubation can affect the health and growth rate of the embryo and in some cases may have lasting effects later in the animal's life (Couch and Ferguson, 1975; McCellan and Novak, 2001).

#### Effects on Offspring Health and Performance.

The majority of studies conducted in poultry were designed to test the effects of supplementing a basal feed (designed to meet the nutritional requirements of the animal) on offspring performance and health (28 out of 34 papers made up the Feeding Method and Nutrition hazard category, a subset of the total 108 papers). For example, carotenoids are thought to confer many benefits including prevention of some infections (Chew and Park, 2004), regulation of cell proliferation and differentiation (Livny et al., 2002), and enhanced antioxidant capacity (Stahl and Sies, 2003). In hens, carotenoids are deposited into the egg yolk and give the yolk its characteristic yellow-orange color (Surai, 2002) but they also provide other benefits to the chick: Feeding broiler breeders carotenoid-supplemented feed led to an increased concentration of carotenoid in the egg yolks (Karadas et al., 2005), improved hatching rates, and enhanced the antibacterial activity of the egg (Cucco et al., 2007), while the immune response in the hatchlings was significantly improved. (Haq et al., 1996).

Supplementing maternal diets with vitamin E (150 and 450 p.p.m.) 2 wk prior to an immune challenge (2 mL intramuscular injection of *Brucella abortus* and Freund's incomplete adjuvant in a 1:1 ratio) increased antibody levels in the plasma of chicks at 2 and 7 d of age; however offspring from hens fed 90, 300, and 900 p.p.m. vitamin E did not exhibit any response. This inconsistent relationship between vitamin E and antibody transfer also has been found in other species (Jackson et al., 1978). Additionally, vitamin E, as well as some minerals, can have beneficial effects on reproductive performance. Taiwan native breeder hens given 40 to 120 mg/kg vitamin E and broiler breeders given 0.5 mg/kg Se in their basal diet had increased fertility and hatchability of offspring (Tsai et al., 2008) and decreased embryonic mortality (Pappas et al., 2006). However, some supplements, like high concentrations of vitamin D_3_ (cholecalciferol) (2,500 to 5,000 μg/kg) or added fats (sunflower or fish oil), resulted in decreased fertility and hatchability compared to low (24 μg/kg) or unsupplemented concentrations (Ameenuddin et al., 1986; Pappas et al., 2006; Bozkurt et al., 2008). Similarly high concentrations of Zinc (150 μg/g) in laying hen diets may cause marginal immunosuppression in the chicks but does seem to affect their growth (Stahl et al., 1989).

Vitamin D_3_ given to broiler breeders can, however, improve leg health in the offspring by decreasing the incidence of tibial dyschondroplasia and decreasing rickets scores, while BW and feed intake increase with increasing supplementation (D_3_: up to 4000 IU/kg) (Atencio et al., 2005a,b; Driver et al., 2006). D_3_ supplementation also can lead to faster morphological gut maturity, and, as a result, function in the offspring when the hens are provided 4,000 IU/kg compared to a control diet of 1,000 IU/kg (Ding et al., 2011). Suboptimal nutrition of the hen and/or the developing embryo can also, unsurprisingly, affect the offspring. Malnutrition during incubation (achieved by removing quantities of albumen from the egg) can lead to raised corticosterone levels, brain sparing through the diversion of well oxygenated blood to the brain at the possible expense of other organs (measured by calculating the mean brain to BW ratio), low hatch rates in the chicks (Rodricks et al., 2004), and decreased post-hatch BW up to 7 d of age (Everaert et al., 2013). However, during the laying phase (18 to 55 wk of age) hens hatched from eggs that had partial albumen removal had higher BW than control and sham removal birds, but they also had reduced egg weights, laying rates, and egg mass and an increased number of second-grade eggs (Willems et al., 2013). Restricting Dwarf broiler breeders to 90% of what they would eat ad libitum resulted in decreased egg production, reduced hatchability, and decreased egg and chick weights compared to ad libitum fed birds (Triyuwanta and Nys, 1992); however when these birds were fed ad libitum, they had reduced fertility rates compared to restricted birds (Triyuwanta and Nys, 1990). A more severe feed restriction (or complete feed withdrawal) also can be used in forced molting to “reset” the reproductive system of birds and results in heavier eggs with better hatchability than the eggs being produced before the molt (Tona et al., 2002), although there is hen mortality and decreased immune function associated with this procedure (Patwardhan and King, 2011).

#### Effects on Offspring Behavior and Cognition.

Maternal nutrition can affect the behavior and cognition of the offspring. For example, laying partridges fed feed supplemented with higher concentrations of n-3 fatty acids (4.40 g/kg) had chicks that performed better in a memory retention task at one d of age than chicks from hens receiving a lower concentration of n-3 fatty acid (0.48 g/kg) (Fronte et al., 2008). Also, the method of feeding itself can have effects on the offspring. Laying hens that were exposed to an unpredictable food restriction schedule had chicks with longer tonic immobility durations, which is thought to be an indicator of fear, than ad libitum fed birds (Janczak et al., 2007a).

Thus there is good evidence that additional supplementation of the hen's feed above the recommended NRC standards with some vitamins or minerals can result in improved growth, development, and/or learning in the offspring. Restricting nutrients has mixed results on the offspring—hatched chicks may be smaller than those of ad libitum fed birds but free feeding of some strains, like broiler breeders, can lead to decreased fertility and hatchability. In addition, there are still a number of unresolved welfare issues surrounding feed restriction of parent stocks (see Decuypere et al., 2010).

### Environmental Parameters

There are a number of environmental or climatic parameters that could affect the offspring, including the pre-lay environment of the hen and the environment that the embryo is exposed to between ovulation and hatching.

#### Effects on Offspring Health and Performance.

Incubation temperature or fluctuations in temperature can have both positive and negative influences on the hatched chicks. For example, increasing the incubation temperature from 37.8 to 39.5°C and the RH from 56 to 65% for 3 h a d during the second wk of incubation (d 8 to 10) increased chick hatchability compared to standard environmental parameters or periodically increasing the temperature and RH during the last wk of incubation (d 16 to 18) (Collin et al., 2007). Eggs exposed for short periods of time (3 h) to increased temperature (to 39.5 or 41°C) later in incubation still had chicks with increased hatchability compared to standard incubation parameters (Yahav et al., 2004). Periodic exposure to cold temperatures (30 min on d 18 and 2 30-min sessions on d 19 of incubation) was also beneficial, resulting in chicks hatching with higher BW than those not exposed to cold stress (Shinder et al., 2009). Embryos that were cooled for 6 h/d from d 15 to 18 also had enhanced thyroxine response to the thyrotrophin releasing hormone, which may help the chick cope with later stressors (Decuypere et al., 1988). However longer exposure to relative extremes of temperature (e.g. temperature reductions to 36.9 to 38°C for 6 h/d for the first 8 d of incubation) led to reductions in hatchability or chicks with decreased BW (Byerly, 1938 in Oppenheim and Levin, 1975; Yalcin and Siegel, 2003). Reduced temperatures (36.7°C) early in incubation also led to broiler chicks with reduced relative shank and femur weight and chicks with more leg problems than those incubated at standard temperatures (Oviedo-Rondon et al., 2009). Additionally, longer exposure to higher temperatures (38.9°C from d 7 of incubation onwards) led to chicks with 26% lower heart weights at hatch and higher overall levels of mortality and ascites-related mortalities (4.1 and 3.8% higher, respectively) than chicks from eggs incubated at the standard temperature of 37.8°C (Molenaar et al., 2011a). It is important to note that some of the temperatures referred to above are invariably the so-called set temperature (the temperature to which the incubator thermostat is set). Early on in incubation, the set temperature and embryo temperature will be similar if not identical; however, once past the mid-time point of incubation, metabolic heat output increases dramatically, meaning that the temperature experienced by the embryo is invariably greater than the set (or air) temperature. This does not alter, however, that there is an interaction between the age of the embryo, the duration of exposure, and the temperature to which the embryo is exposed, with the optimum conditions varying depending on the parameter selected.

In addition to incubation temperatures, the temperatures that the hens are housed at also influences offspring characteristics. Hens kept during wk 22 to 27 of life under standard thermal conditions (21°C) or warmer but within the thermotolerance range conditions (31°C) had similar laying rates. However, the hens raised in warmer temperatures had lighter eggs and higher concentrations of yolk progesterone, testosterone, and estradiol, and chicks hatched from these eggs had higher morphological quality scores (based on measures described in Tona et al., 2003) that varied from zero (very bad) to 40 (very good) and made fewer distress calls when exposed to a novel food. These findings may indicate a beneficial adaptive response as maternal environment can result in different offspring phenotypes that may be more suited to particular environmental conditions (Bertin et al., 2013).

Factors such as the age of the parent flock will affect the offspring. Generally younger birds have smaller eggs that result in lighter offspring, chick weights in poultry species typically being approximately 70% of the total egg weight. For example, broiler chicks hatched from young parents (27 or 32 wk of age) had lower BW than chicks hatched from older parents (57, 42, or 65 wk old) (Nelson et al., 1992; Yalcin et al., 2008), and quail chicks from younger parents (11 wk of age) were lighter than chicks from older parents (37 wk of age) at hatch (Guibert et al., 2012). Additionally, eggs from younger (24 wk of age) hens had higher yolk levels of antibody Immunoglobulin Y (**IgY**) than birds from 36- and 82-week-old hens (Barua et al., 2000), which may have implications for disease control programs. However, broiler breeders just coming into lay (about 23 wk old) had lower egg fertility and hatchability and higher embryo mortality, indicating that there is a lower limit to the benefits of incubating eggs from young-parent birds (Pappas et al., 2006).

Egg storage times before incubation also can have effects on the subsequent offspring. Fertilized eggs are typically stored for approximately 7 d before being incubated. Eggs stored for longer periods (18 d before incubation) had delayed hatching and an increase in triiodothyronine (**T3**) most likely due to higher concentrations of corticosterone between internal pipping and hatching and which may indicate a more stressful hatching event compared to embryos hatching from eggs stored for 3 d (Tona et al., 2003). Embryos stressed due to hypoxia (O_2_ decreased below physiological concentrations) and anoxia (total depletion of O_2_) also resulted in increased plasma corticosterone levels in newly hatched chicks and had an increased number of adverse conditions such as a delay in righting response (Rodricks et al., 2008), impaired development of thermogenesis (Azzam et al., 2007), and lower BW for up to 10 d post-hatch (Camm et al., 2001) compared to embryos that did not experience hypoxia during incubation. Chicks exposed to high O_2_ levels (25%) during d 9 to 19 of incubation also had decreased residual yolk weight at hatch and higher yolk-free weights and longer chick body lengths than chicks incubated with lower (21%) O_2_ concentrations (Lourens et al., 2007). Yolk-free body mass at d 18 of incubation was also higher with higher O_2_ levels, although differences between high and normal O_2_ concentration treatments disappeared by 48 h after hatch (Molenaar et al., 2011b). Fertile eggs exposed for 24 h to 6% CO_2_ and either 10 or 20% O_2_ during the first 10 d of incubation had increased cardiac and non-cardiac malformations, such as thickening of the inter-atrial septum demonstrating that increased CO_2_ levels can be teratogenic; however, newly hatched chicks were also 10% heavier than in the control groups (Haring et al., 1970).

Exposure to environmental toxins, which would be more prevalent among wild or farmed birds with access to range, or which could contaminate crops that are made into feeds for farmed animals, usually had negative effects in the offspring. 2,3.7,8-tetrachlorodibenzo-p-dioxin (**TCDD**) is a toxin known to cause, among other symptoms, wasting syndrome, carcinogenicity, and immunotoxicity (Poland and Knutson, 1982). It can get into the environment through combustion and incineration and chemical and biological processes. Poultry were used as a model for wild turkeys to determine the risks of exposure. Chicks from eggs injected with the toxin showed suppressed B-cell proliferation and catalytic enzyme production (Peden-Adams et al., 1998). One-time exposure of Japanese quail embryos to dichlorodiphenyldichloroethylene (**DDE**) resulted in female offspring with accelerated puberty onset and male offspring with reduced reproductive performance (e.g. mount attempts); however, reproductive physiology in adults appeared to be unaffected (Quinn et al., 2008). This means there may be immune and neurological risks for the offspring of wild or farmed birds that come into contact with the external environment or that are fed contaminated feedstuffs, especially if exposure occurs when young.

#### Effects on Offspring Behavior and Cognition.

Increasing incubation temperature also can have positive behavioral effects: Chicks from eggs exposed to 24 h of 40.6°C incubating temperature on d 16 showed less aggressive pecking to conspecifics at 21 wk of age than those from eggs incubated under standard conditions, although it is not clear exactly what the mechanism behind this difference in behavior was (Lay and Wilson, 2002). However, chicks from eggs exposed to hypoxic conditions (24 h of 14% O_2_) on d 10 or d 14 of incubation had impaired short-term memory but this was not consolidated into long-term memory impairment (Gibbs et al., 2009). Additionally, chicks from eggs that were incubated with decreased O_2_ levels during d 10 to 14 were less successful in an avoidance discrimination task (exposed to 2 different color beads: one had an aversive taste; the other did not) than birds hatched from control eggs (Rodricks et al., 2004), and chicks from eggs partially wrapped in a membrane during incubation also had reduced success in an object avoidance test 60 and 120 min after being taught the task (Camm et al., 2001).

Offspring from eggs exposed to 2 h of pulsing bright lights (750 to 1,000 lux) on d 18 of incubation, feather pecked more on d 7, 14, and 21 after hatch (Riedstra and Groothuis, 2004). The authors hypothesized that this was due to light exposure influencing lateralization of the brain, thereby decreasing the ability of the birds to discriminate between familiar and unfamiliar birds whom they would “explore” by pecking. Conversely, embryos incubated with a 12L:12D light schedule had better retention of a passive avoidance and discrimination task after hatch compared to dark-incubated only chicks (Sui and Rose, 1997). Occasional exposure to sounds in the incubation environment (15 min/h from d 10 to hatching), using both natural, species-specific noises and artificial noises (sitar music) increased hippocampal function (Chaudhury et al., 2009), which may facilitate learning and memory, and influenced postnatal auditory preferences (Jain et al., 2004); however, only high-decibel music (110 dB) positively modulated spatial orientation, learning, and memory when compared with noise (non-patterned or rhythmic) of the same decibel levels (Sanyal et al., 2013). Maternal age also can influence offspring characteristics: Chicks from 37-week-old Japanese quail were less reactive when encountering a novel environment, but they were more stressed by social isolation than chicks from 11-week-old quail (Guibert et al., 2012).

Stress also can affect the offspring through inherited epigenetic modifications and this process may have been favored by domestication: White Leghorn and Red Junglefowl hens undergoing stress in the form of unpredictable light:dark rhythms showed decreased ability to perform a spatial learning task; however, this effect was greater in the White Leghorn hens. These White Leghorns also had offspring with reduced spatial learning abilities that were more competitive and that grew faster than offspring from non-stressed hens, whereas offspring from the Red Junglefowl hens exposed to the same stressors did not exhibit this reduction in spatial learning (Lindqvist et al., 2007).

Thus, small environmental manipulations, such as short increases or decreases in incubation temperature can have beneficial effects on the offspring while young to middle-aged parent flocks tend to have more successful offspring than very young or old parent flocks. Irrespective of the age of the parent bird, storage times that exceed 7 d (e.g. 18 vs. 7 d storage), varied lighting schedules, restricted gas exchange across the egg shell, and exposure to toxins may lead to detrimental effects on the offspring and should be avoided when possible.

### Artificial Parameters

Artificially manipulating the concentration of stress hormones in the individual or their offspring can give an insight into the potential effects of any related stressor.

#### Effects on Offspring Health and Performance.

Corticosterone is the most frequently studied stress hormone in poultry but other hormones (such as noradrenaline) or lines of birds selected for low or high stress levels are also studied. For example, quail selected for high plasma corticosterone laid eggs with higher yolk corticosterone (Hayward et al., 2005) and had lower egg fertility (Schmidt et al., 2009b) than birds selected for low plasma corticosterone. The offspring from “high stress” quail had greater size differences between their left and right tibia and middle toe lengths, indicating that developmental instabilities like increased stress can lead to greater fluctuating asymmetry in the offspring (Satterlee et al., 2008). Raised corticosterone levels in hens (achieved by corticosterone implants or injections) also decreased growth rates and increased mortality in their chicks compared to hens with non-functional implants or injected with saline (Hayward and Wingfield, 2004; Hayward et al., 2006; Satterlee et al., 2007; Wall and Cockram, 2010), and eggs with increased corticosterone, e.g. from corticosterone injections, led to increased mortality and decreased BW in the chicks for up to 77 d post-hatch (Eriksen et al., 2003; Janczak et al., 2006, 2007b). Quail subjected to corticosterone injections in ovo showed higher corticosterone concentrations when stressed at 64 d of age by being placed individually in an opaque box than quail subjected to sham injections or post-natal corticosterone injections, demonstrating the potentially longer-lasting effects of early stressors (Marasco et al., 2012). Corticosterone also can be raised by subjecting the birds to unpredictable stressors. Japanese quail hens were exposed to mild stressors (unpredictability in the environment and sudden movements) 3 times a d for 24 consecutive d and compared with hens in which no stressors were applied. Stressed hens had heavier eggs, contained more albumen, hatched earlier, and were heavier at hatching than non-stressed hens’ eggs (Guibert et al., 2011). Even the physiology of the parent birds can affect corticosterone levels in the offspring, with chicks hatched from a standard heavy broiler breeder line having higher corticosterone levels than an experimental lighter broiler breeder line (Tona et al., 2004).

Other hormones affect offspring development as well. So, for example, quail eggs injected with testosterone had a lower hatchability and cell mediated immunity than eggs not injected or injected with the carrier only (Daisley et al., 2005; Cucco et al., 2008), although these offspring did have higher growth rates (Cucco et al., 2008). However the negative effects of testosterone supplementation were found to be reduced when chicks were supplemented with carotenoids in their feed (Cucco et al., 2008). Additionally, eggs injected with noradrenaline to increase the basal concentration produced chicks with raised levels of circulating corticosterone (Gibbs et al., 2009).

#### Effects on Offspring Behavior and Cognition.

Early exposure to stress hormones can affect fear behavior. Hens selected for “low stress” had offspring that took longer to induce tonic immobility (**TI**: an indicator of stress or fear, Davis, 2008), and chicks from eggs injected with testosterone had longer TI durations than chicks from eggs that weren't injected with testosterone during incubation (Okuliarova et al., 2007). Eggs injected with corticosterone before incubation resulted in: chicks that had higher latencies in a human-approach test and fewer chicks that would cross a barrier to reach food (Janczak et al., 2006); chicks that had raised basal plasma corticosterone during a beak-trimming stress test (Lay and Wilson, 2002; Rodricks et al., 2006); and chicks that performed more aggressive pecking to their conspecifics (Lay and Wilson, 2002). Chicks from quail exposed to unpredictable mild stressors, such as sudden movements or noises, had higher reactivity when introduced to a novel environment and they reacted more strongly to social isolation (Guibert et al., 2011). However, quail chicks from eggs injected with testosterone had reduced numbers of total calls and a greater latency to call in open field tests, took less time and had a shorter latency to approach novel objects, and had lower fecal corticosterone after a 90-min isolation test than offspring from control eggs (Daisley et al., 2005). Male quail injected with testosterone in ovo showed increased food competitiveness and had a more female-like phenotype than sham treated males, suggesting that maternal androgens interact with sexual differentiation of the brain and behavior and development of secondary sex characteristics (Riedstra et al., 2013).

Corticosterone injections in eggs also influenced learning ability, with chicks from eggs injected with larger doses (0.3 nmol/egg) performing worse in an object discrimination task than those injected with very small doses (0.01 nmol/egg) (Rodricks et al., 2006). Quail chicks injected with noradrenaline and exposed to a maternal call during incubation did not show a preference for the call while chicks exposed only to the call during incubation did (Markham et al., 2006). Similarly, chicks injected with corticosterone during incubation and exposed to an imprinting stimulus (another chick) did not prefer the stimulus, while the control chicks did (Nordgreen et al., 2006).

Thus, there is good evidence indicating that early life exposure to increased stress levels can have a later effect on chick mortality, development, stress sensitivity, and behavior, and this has implications for the management of captive breeder birds and potentially also wild birds. Although stress levels were raised artificially in these studies, other aspects of the production environment can lead to increased stress levels (e.g. Parsons, 1990) and this could affect production and bird welfare.

### Husbandry Parameters

There was only a small sample of studies that examined the effects of husbandry procedures on the offspring.

#### Effects on Offspring Health and Performance.

One study involved the practice of forced molting, a method still practiced in many countries like the United States but is illegal in the United Kingdom and can involve withdrawing food completely from the hens until they have ceased laying for a few d resulting in a wk or more without food (Patwardhan and King, 2011). This practice is stressful for the hens and leads to increased concentrations of antibodies, such as levels of IgY, in the yolks of their eggs for at least 10 d after the hens begin to re-lay, which could have beneficial effects for the early immune function of fertilized eggs (Barua et al., 2001). Even non-invasive procedures such as habituating quail to humans by increased exposure to humans, gentle handling, and food rewards and then exposing habituated and non-habituated quail to human disturbances had significant effects. Eggs had more yolk, lighter shells, increased progesterone, and decreased androgen levels, and the chicks hatched earlier and weighed more than non-habituated quail eggs (Bertin et al., 2008).

#### Effects on Offspring Behavior and Cognition.

Chicks from quail habituated to humans had a greater fear response as measured by tonic immobility tests (reduced number of inductions) and showed more fear behavior when exposed to the experimenter and a novel environment. It was postulated that the habituation process may have been stressful for the hens and may have led to higher stress hormone levels being transferred to the eggs (Bertin et al., 2008). There were also social effects of the habituation treatments as offspring from non-habituated quail spent less time near conspecifics and males showed less crowing and courtship behavior toward females than offspring from habituated quail (Bertin et al., 2009).

The egg quality and social and sexual changes in the offspring have implications not only for birds hatched in production systems but also when interacting with wild species during conservation efforts.

### Other Parameters

Other parameters, such as social environment, housing conditions, and the infectious environment and maternal health status may influence factors in the offspring.

#### Effects on Offspring Health and Performance.

Dominant hens allocate more testosterone to male eggs than female eggs, while subordinate hens allocate more testosterone to female eggs (Muller et al., 2002). Housing environment also can influence the hormones allocated to eggs. Hens housed in floor pens laid eggs with yolks higher in androstenedione and estradiol while caged hens had eggs with higher concentrations of estradiol in the albumen (Janczak et al., 2009).

Vaccination of the hens before they begin to lay can lead to long-lasting maternal antibodies being passed into the egg and offspring. For example, pre-lay hens vaccinated against *Salmonella* led to long-lasting *Salmonella*-specific antibodies found in the egg yolk as Immunoglobulin G (I**gG**) and in the offspring as IgG in the serum and IgA in the intestinal fluid. Chicks from vaccinated hens also had higher antibody response when orally infected with *Salmonella* strains than non-vaccinated hen offspring (Hassan and Curtis, 1996). Vaccinating adult birds and the use of chicks from vaccinated hens are helping to control and reduce infections in the birds from bacteria that can be found in a production environment.

#### Effects on Offspring Behavior and Cognition.

Maternal androgens increase competitiveness in the nestling and aggression and growth rate in the juvenile (Schwabl, 1993, 1996; Eising et al., 2001). These hormones may be a way for the hen to signal the state of the environment to her offspring and subsequently affect their development and phenotype. Also, prenatal sensory experience has a role in the lateralized postnatal visual discrimination in birds. During the hatching process, the chick embryo has its left eye blocked by its body and yolk sac but the right eye is exposed to light coming through the shell, leading to a 90% left side turning bias found in domestic chicks (Rogers, 1991). However, when both eyes are prenatally exposed to light or when the left eye is exposed and the right eye is blocked from light, the turning bias disappears, while birds who experimentally had their right eyes exposed and left eyes blocked from light prenatally still maintained the turning bias (Casey and Karpinski, 1999). Thus treatment of incubating and hatching eggs can have long lasting effects on the brain and behavior of avian offspring.

## DISCUSSION

There are numerous factors that can have an influence on the hen and the incubating egg that will result in lasting effects on the phenotype of the offspring. These can range from the supplements provided in the feed (e.g. Atencio et al., 2005a,b) or even the amount of feed provided (e.g. Triyuwanta and Nys, 1992), the temperature and relative humidity during incubation (e.g. Collin et al., 2007), to age of the parent flock (e.g. Yalcin et al., 2008), levels of stress hormones in the hen (e.g. Schmidt et al., 2009a,b), habituation of the parents to humans (e.g. Bertin et al., 2009) to the dominance status of the hen (e.g. Muller et al., 2002). Some factors can result in benefits to the offspring, such as supplementing vitamin D_3_ in broiler breeder feed (Driver et al., 2006) while others may have detrimental effects, such as stress in the parents, leading to a reduction in memory and learning in the offspring (Rodricks et al., 2006).

The presence of some factors, like exposure to environmental toxins (e.g. Quinn et al., 2008), is unlikely to occur in current commercial poultry production where the birds are housed, although the trend to move towards extensive systems has the potential to increase the risks (Schoeters and Hoogenboom, 2006). However there is a risk that farmed poultry may be fed feed made with crops exposed to environmental toxins (van Barneveld, 1999). Other factors, like restricted feeding in broiler breeders, are standard practice (e.g. Triyuwanta and Nys, 1992). The most prevalent result of the different hazards found in production systems is likely to be increased stress leading to increased concentrations of circulating corticosterone. Increased stress could be caused by parasitic infections, pathologies, feed deprivation, social stress, exposure to novelty, and noise and behavioral restrictions (e.g. Parsons, 1990), meaning that the standard housing of poultry could be contributing to raised corticosterone levels compared to less intensively reared or wild fowl. This increased stress could lead to offspring with decreased weights, increased mortality, learning impairments, and increased stress sensitivity (e.g. Hayward and Wingfield, 2004). However more research is needed to determine how stressful current systems are in terms of, for example, elevated concentrations of corticosterone. Flock age is an ongoing concern in poultry production, as breeder birds may be placed in systems pre-lay and continue producing for well over a yr. Older flocks have chicks with higher BW, but they also have higher chick mortality (Yalcin et al., 2008) and lower yolk antibodies (Barua et al., 2000), which can have implications for the total number of viable offspring produced. Some deleterious effects are commonly encountered, such as reduced porosity in eggs from young breeding birds, whereas stressors such as bright pulsing lights during incubation would occur only in accident or emergency situations and should not present a regular problem for poultry welfare.

However, some hazards, like periods of increased or decreased temperatures during incubation, can be beneficial and may more closely mimic a hen brooding a clutch of eggs who takes breaks from sitting (White and Kinney, 1974). Supplementing poultry feed is a relatively easy way to provide additional protection to the offspring, e.g. vitamin D_3_ given to broiler breeders resulted in chicks with better leg health (Atencio et al., 2005a,b) but dosages should be monitored to prevent any negative effects, e.g. high levels of zinc being supplemented to hens acted as a mild immunosuppressant in the chicks (Stahl et al., 1989), although it should be noted that the studies examining these effects have been fairly small-scale experimental trials to date. On-farm studies would be useful to confirm that the same positive effects occur on a much larger scale and that changing the factors, such as incubation temperatures, is practically and economically feasible.

In terms of poultry welfare measures, offspring condition could reflect the suitability of the parent environment. For example, it has been suggested that fluctuating asymmetry in chicks be used as a proxy measure for chronic stress in the hens as asymmetries increase with increased stress (Satterlee et al., 2000). So hens in one environment producing chicks with larger asymmetries than hens from another environment may be more stressed and have poorer welfare. The differences in the 2 environments can then be compared to attempt to improve conditions for the more stressed hens. Other measures such as comparing chick mortality, growth rate, leg conditions, etc., from hens being housed and managed in different ways could also be used to assess suitability of the hen environment. Using offspring to assess parent environment has the advantage that the animals in the environment under question are not interfered with; however, more research and validation studies using this method compared to other generally accepted welfare measures are needed before offspring measures become a widespread parent welfare measurement.

In conclusion, the maternal environment includes many factors such as feeding method and nutrition, environmental parameters, artificial challenges, husbandry, housing system, social environment, infectious environment, and maternal health status, and these all can influence the development, physiology, and behavior of the offspring. As the conditions we keep commercial poultry in are quite artificial compared to their wild ancestors, they have the potential to be stressful, which could negatively affect the offspring. The literature strongly suggests that maternal conditions prior to lay, or conditions during incubation could have important effects on poultry health, welfare, and productivity. Consideration of how potential negative prenatal effects can be avoided may benefit poultry production. Additionally, we also have the potential to provide factors to the hen that will benefit the offspring and help improve their welfare. Using the offspring to help assess suitability of parent environment is a promising welfare assessment tool but it requires further study and validation against current welfare measures before becoming a widespread method.
